# Concurrent Validity of Isokinetic Hip and Knee Strength Measurements Using the UGO Exoskeleton in Healthy Young Adults: Cross-Sectional Validation Study

**DOI:** 10.2196/99564

**Published:** 2026-10-05

**Authors:** Hao Xie, Ming-Hui Ding, Pei-Ming Chen, Shamay S M Ng, Chu-Huai Wang, Jiang-Li Zhao

**Affiliations:** 1 Department of Rehabilitation Medicine, the First Affiliated Hospital of Sun Yat-sen University Guangzhou, Guangdong China; 2 Department of Rehabilitation Sciences, the Hong Kong Polytechnic University Hong Kong China (Hong Kong); 3 Department of Rehabilitation Medicine, Guizhou Hospital of the First Affiliated Hospital of Sun Yat-sen University Guiyang, Guizhou China

**Keywords:** exoskeleton rehabilitation robot, isokinetic strength testing, peak torque, validity, lower extremity

## Abstract

**Background:**

The UGO lower limb exoskeleton rehabilitation robot is designed for patients with lower limb motor dysfunction, and it can be used to assess the peak torque of hip and knee flexors and extensors.

**Objective:**

The primary aim of this study was to establish the concurrent validity of peak isokinetic torque using the UGO exoskeleton and Humac Norm dynamometers in healthy young adults.

**Methods:**

Twenty healthy young adults were enrolled in this study. All participants underwent isokinetic muscle strength tests using both the UGO exoskeleton and the Humac Norm dynamometers. Peak torque (Nm) of the hip and knee flexors and extensors was measured. The Pearson correlation coefficient was used to determine concurrent validity. Bland-Altman analysis was used to assess the agreement between the 2 devices.

**Results:**

At the knee, peak torque of the extensors measured by the UGO exoskeleton correlated strongly with that measured by the Humac Norm dynamometer (right: *r*=0.662; *P*=.001; left: *r*=0.768; *P*<.001), with 95% limits of agreement (LoA) ranging from −22.89 to 82.21 Nm and from −24.60 to 75.65 Nm, respectively; the right knee flexors showed a moderate correlation (*r*=0.547; *P*=.01), with 95% LoA ranging from −26.27 to 36.01 Nm, whereas the left knee flexors showed no significant association (*r*=0.347; *P*=.12), with 95% LoA ranging from −2.40 to 75.02 Nm. At the hip, peak torque of the flexors correlated strongly on the right (*r*=0.615; *P*=.003) and moderately on the left (*r*=0.443; *P*=.04), with 95% LoA ranging from −15.52 to 86.57 Nm and from −12.53 to 98.50 Nm, respectively, whereas the bilateral hip extensors showed no significant association (right: *r*=0.337; *P*=.14; left: *r*=0.391; *P*=.20), with 95% LoA ranging from −51.57 to 76.80 Nm and from −28.19 to 79.59 Nm, respectively.

**Conclusions:**

In healthy young adults, the UGO exoskeleton demonstrated moderate to strong concurrent validity for peak torque of the bilateral knee extensors and hip flexors, but not for the nondominant knee flexors or bilateral hip extensors. The device may serve as a convenient torque assessment tool for selected muscle actions, but systematic overestimation and posture-related differences must be considered, and validation in the intended clinical population of patients with neurological conditions is required in further studies.

## Introduction

Lower limb motor dysfunction is a significant complication associated with normal aging or nervous system diseases that may result in a considerable decline in quality of life [1-3], impose a heavy health burden, and lead to substantial economic losses to society [4]. Lower limb muscle strength is an important factor in maintaining balance and gait [5,6]. Among lower limb muscles, knee extensors and flexors play a critical role in weight bearing, propulsion [7], and stability during ambulation [8], making their accurate assessment essential for rehabilitation planning and outcome evaluation. Traditional muscle strength testing methods, such as manual muscle testing, are widely used in clinical settings due to their simplicity and low cost. However, these methods suffer from well-documented limitations, including subjective interpretation, assessor strength bias, and poor reliability [9], particularly when testing stronger muscle groups such as the knee extensors in individuals who are healthy or moderately impaired. Therefore, the ability to assess muscle strength efficiently and with high precision has become a clinical imperative. Isokinetic dynamometers are known as the gold standard for muscle strength testing because of their high accuracy. However, their clinical utility is limited by their cumbersome design, stationary nature, and high cost [10].

Recently, exoskeleton-based rehabilitation robotics has emerged as a cutting-edge intervention for restoring function in patients with neurological sequelae [11-13]. A hallmark of these systems is the capacity to autonomously deliver highly repeatable, standard rehabilitation exercises, dramatically reducing the physical load on therapists [14]. Exoskeleton rehabilitation robots have emerged as a promising intervention for neurological recovery. Current hot topics include upper limb goal-directed training, lower limb motion control strategies, and exoskeleton rehabilitation robot power [15]. A pilot randomized controlled trial of the REX robotic exoskeleton (REX Bionics PLC), a self-stabilizing lower limb device that supports patients in a hands-free upright standing position without crutches or walking frames, included 3 supplementary training programs—standing balance activities (pushing a Bobath ball and multidirectional reaching), thera-band elastic band resistance training following an upper limb PNF diagonal spiral pattern, and lower limb function training (single-leg weight bearing, lateral and repeated strides, squatting, and alternating strides)—to promote balance and lower limb motor recovery in patients with subacute stroke [16]. The integration of series elastic actuators in lower limb exoskeletons has significantly advanced robotic rehabilitation, particularly for enhancing human-robot interaction safety and motion control [17]. Prasad et al [11] proposed 4 conceptual configurations for cable-driven exoskeletons, which address key power-related challenges inherent in traditional actuation systems.

Exoskeleton rehabilitation robots overcome some limitations of traditional clinical assessment by providing more objective, sensitive, reliable, and efficient measurements [18]. Several groups have proposed using robotic devices to assess proprioception and motor function [18-20], such as gait and joint position sense; the Lokomat, for example, has been used to assess lower limb joint position sense [19], and the bilateral Harmony upper limb exoskeleton can measure joint angles across the shoulder, forearm, and wrist [21]. An intelligent assessment study using a lower limb exoskeleton for patients with stroke reported predictive accuracies of 85% for the 6-minute walk distance and 93% for the Fugl-Meyer assessment lower extremity score, outperforming traditional manual measurement in efficiency and objectivity [22]. Although these studies establish that exoskeleton and robotic platforms can quantify kinematic and functional variables, the agreement of an exoskeleton’s isokinetic torque output with a criterion standard dynamometer has, to our knowledge, not been reported. The UGO rehabilitation exoskeleton is seeing growing adoption in clinical practice for treating patients with lower limb motor dysfunction resulting from stroke, spinal cord injury, or peripheral nerve injury [23]. Designed to provide high-frequency, repetitive, standardized gait training, the device additionally features isokinetic muscle strength testing as a key diagnostic function. Unlike conventional isokinetic dynamometers, it is equipped with force sensors at both the hip and knee joints on each side, so that bilateral testing does not require positional changes—a design that theoretically makes testing more convenient and efficient. Although the UGO exoskeleton is widely used and has recognized theoretical benefits, the measurement properties of its strength assessment capability remain to be established. These properties are conventionally established in 2 distinct steps. In a previous study, we examined the intratester and intertester reliability of the device (an internal, device-only property—whether it produces reproducible values) [24]. The present study addresses the separate and complementary question of concurrent validity (an external property—whether the device agrees with an accepted reference standard). Reliability is a prerequisite for, but is conceptually and statistically independent of, validity: a device may be reliable yet invalid. The 2 studies drew on an overlapping healthy participant pool but used entirely different analyses and report no duplicate results; the present work provides the first agreement-based, reference standard comparison of the UGO’s isokinetic torque function.

The aim of this study was to determine the concurrent validity of isokinetic hip and knee peak torque measurements obtained with the UGO rehabilitation exoskeleton against those obtained with the Humac Norm dynamometer in healthy young adults, using both agreement and association analyses. We hypothesized that peak torque measured by the 2 devices would show at least moderate, statistically significant correlations and clinically moderate to strong agreement for the major muscle actions.

## Methods

### Study Setting

This study was carried out in the Biomechanical Rehabilitation Engineering Laboratory of the Department of Rehabilitation Medicine at the First Affiliated Hospital of Sun Yat-sen University. Participants were randomly recruited from interns within the department. Measurements were taken by the same physical therapist, who had >5 years of clinical experience and was proficient in operating both pieces of equipment, using the 2 isokinetic dynamometers. This tester had previously undergone training and passed evaluations related to the operation of isokinetic muscle strength testing equipment for both devices.

### Sample Size Calculation

According to a previous study [25] that investigated the correlation between a Biodex isokinetic dynamometer (Evome Medical Technologies Inc) and a hand-held dynamometer in male football players (*r*=0.322-0.617), a sample of 17 participants provides approximately 80% power to detect a correlation of *r*=0.60 at α=.05 (2-tailed) in this study, as calculated using G*Power. To draw a stronger conclusion, the sample size was finally determined to be 20.

### Recruitment

This study recruited participants via 2 approaches. First, a recruitment announcement was posted in the WeChat (Tencent Holdings) group for interns within the department, followed by on-site screening to identify eligible candidates. Baseline information such as age, sex, height, weight, BMI, and the dominant lower limb were collected for each participant. Researchers contacted potential participants who satisfied the testing equipment requirements (height between 150 cm and 190 cm, weight not exceeding 100 kg) to confirm their willingness to participate [23].

### Inclusion Criteria

The inclusion criteria were as follows: (1) aged 20 to 30 years, (2) absence of any history of cardiovascular or respiratory diseases, (3) lack of a history of lower limb musculoskeletal conditions (eg, hip or knee joint surgery, ligament injury, or muscle strain or sprain) that would affect the test, and (4) absence of high-intensity physical activities during the past week.

### Ethical Considerations

The study was approved by the medical ethics committee of the First Affiliated Hospital of Sun Yat-sen University in Guangzhou, China ({2020}430) and conducted according to the Declaration of Helsinki. This was a safe and noninvasive experiment. Prior to the commencement of the experiment, a member of the research team elucidated the experimental procedures to all participants and obtained their informed consent. Furthermore, throughout the experiment, the participants had the option to withdraw from the experiment at any time without providing any reason.

### Instrument

Two isokinetic dynamometers were used in this study: the UGO rehabilitation exoskeleton (Chengtian) and the Humac Norm (CSMi Solutions) (Figure 1). The UGO lower limb exoskeleton robot is widely used as a research and clinical platform for validating gait planning algorithms. Notably, its capacity for isokinetic concentric torque testing at the hip and knee joints constitutes one of its most important evaluation functions, providing reliable and functionally relevant measurements in a natural upright posture. Many studies have confirmed that the Humac Norm is a highly reliable and accurate isokinetic dynamometer [10,26-28]. Identical angular velocity (60°/s) and joint range-of-motion settings were configured on both dynamometers; body posture, however, differed between the devices by design (UGO: upright; Humac Norm: seated for the knee, supine for the hip).

**Figure 1:**
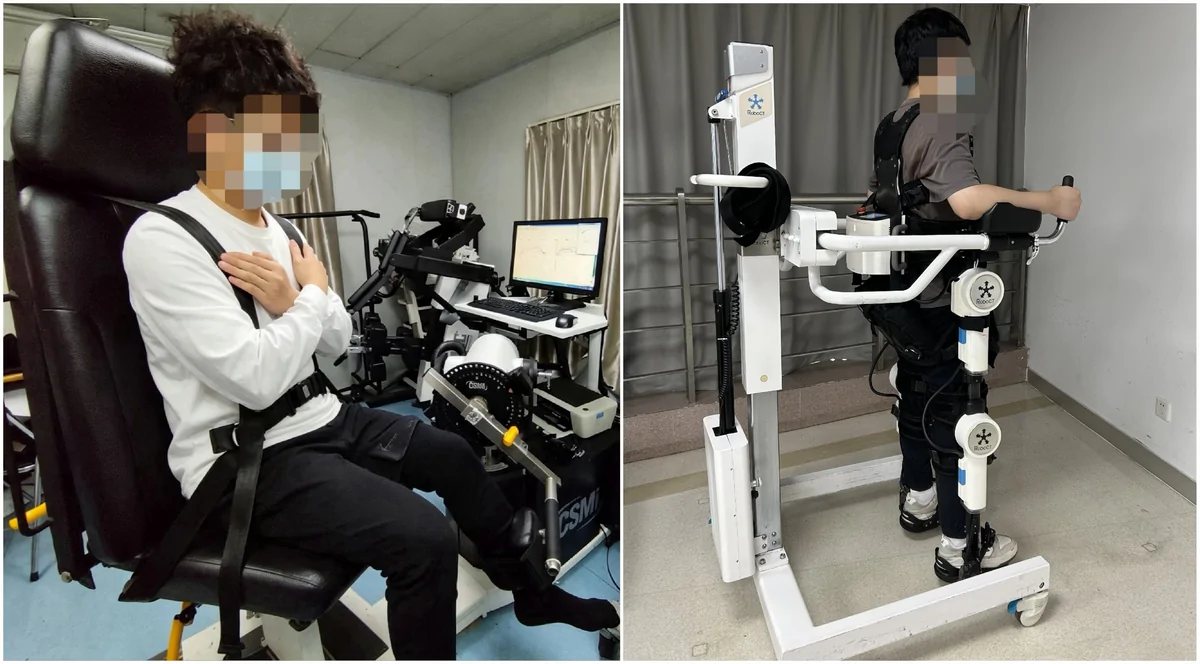
Isokinetic muscle strength testing of participants using the UGO rehabilitation exoskeleton (Chengtian) and Humac Norm isokinetic dynamometer (CSMi Solutions).

### Parameter

This study primarily focused on collecting peak torque data for the knee and hip flexor and extensor muscle groups during concentric contractions using the 2 isokinetic dynamometers. The isokinetic dynamometers measure muscle strength by collecting mechanical signals within the range of motion through sensors and calculating the measurements using their respective analysis software. The parameter that was recorded was absolute peak torque (in Newton-meters). Peak torque has been defined as the maximum force produced by a muscle during voluntary contraction within the range of motion under a given set of conditions [29,30].

### Procedure

To minimize the potential influence of biological rhythms on the results, the 2 tests were conducted at the same time on 2 consecutive days. Participants were instructed to abstain from strenuous physical activity and refrain from consuming any nutritional supplements or functional beverages that could enhance physical performance, starting 48 hours before the tests and continuing until their completion. They were permitted to maintain their usual daily activities and diet. Prior to the formal test, participants performed 3 minutes of lower limb muscle stretching, followed by 10 knee and hip flexion and extension repetitions at a submaximal voluntary contraction level to familiarize themselves with the testing procedure. After the warm-up, each participant rested for 5 minutes to prevent fatigue. The testing sequence involved assessing the dominant side first, followed by the nondominant side after a 2-minute rest, and the knee and hip tests were performed 5 minutes apart.

To ensure consistency and comparability, in the knee test, both devices used the lateral femoral condyle as the axis of motion, with a movement speed of 60°/s and a range of 0° to 70° (0°: the fully extended position of the knee joint) for knee testing. For the UGO device, thigh and calf lengths of participants were adjusted so that the axis of movement was aligned with the lateral femoral condyle in a seated posture, and their trunk, distal femur, proximal tibia, and foot were securely fixed to avoid compensatory forces. Testing was performed in an upright position, with the external frame fixed first, and the muscle strength test of the flexion muscle group was started from the extension position of the knee joint. Each movement was followed by a cue tone, followed by movement in the opposite direction. Verbal instruction for peak torque was provided as “flexion and extension as hard and as fast as you can” to ensure maximal effort during each action.

The greater trochanter of the femur was used as the axis of motion, with a movement speed of 60°/s and a range of −10° to 60° (0° being the neutral position of the hip) in hip testing. The position was the same as that for knee testing and started from the extension position. During the UGO isokinetic muscle strength test, repositioning was not required when switching between test sides or joint angles, which saved therapists both time and operational steps. The bilateral test took 5 minutes to complete for each joint, during which 5 consecutive flexion and extension movements were executed, and peak torque values were recorded after each movement.

In accordance with the standardized testing protocol of Humac Norm, participants were seated on the isokinetic dynamometer with their hip joints flexed at 85°. The tension sensor’s rotation angle was set at 40°, and its height was adjusted to 8 cm. The rotational axis of the knee was placed in line with the dynamometer axis of rotation, and 0° was determined as 0° knee extension. The resistance arm pad was secured just proximally to the medial malleolus, so that movement of the ankle was not constricted. To minimize compensatory trunk movements, straps were used to secure the participant’s trunk, distal femur, and distal tibia. During the test, participants were required to cross their arms in front of their chest to prevent compensatory force generation by gripping the seat. The hip test was performed in the supine position; the tension sensor’s rotation angle was set at 0° and its height was set at 9 cm. The resistance arm pad was secured just distal to the femur, and a fixation band was used to fix the pelvis. Upon program initiation, 5 consecutive flexion and extension movements were completed.

### Statistical Analysis

Descriptive statistics were used to analyze the demographic characteristics of the participants in this study. A 1-sample Kolmogorov-Smirnov test was used to test whether the data were normally distributed. The Pearson correlation coefficient (*r*) or Spearman rank correlation coefficient (ρ) was used to determine concurrent validity. Here, *r* or ρ values between 0.00 and 0.19, between 0.20 and 0.39, between 0.40 and 0.59, between 0.60 and 0.79, and ≥0.80 represented very weak, weak, moderate, strong, and very strong correlations, respectively [31]. A scatter diagram was constructed to evaluate agreement between the UGO and HUMAC measurements and to identify any systematic bias [10]. A Bland-Altman plot was used to compare the mean difference and the 95% limits of agreement (LoA) between the 2 devices. The upper and lower 95% LoA were calculated as the mean difference±1.96×SD. All statistical analyses were performed using SPSS (version 20.0; IBM Corp), with a significance level set at *P*<.05.

## Results

### Overview

Twenty healthy young adults were recruited for this study. All participants cooperated well during the tests, and each participant exerted maximal effort. The demographic characteristics of the participants are provided in Table 1. Of the 20 participants, 9 (45%) were male, 11 (55%) were female, and all 20 (100%) were right-lower-limb dominant.

**Table 1:** Demographic characteristics of the study participants (N=20).

Variables			Values
Age (years), mean (SD; range)			22.70 (0.98; 21.0-25.0)
Height (cm), mean (SD; range)			167.65 (7.87; 156.0-180.0)
Weight (kg), mean (SD; range)			62.08 (12.16; 47.0-87.0)
BMI (kg/m^2^), mean (SD; range)			21.90 (2.75; 18.6-28.4)
**Sex, n (%)**			
	Male	9 (45)	
	Female	11 (55)	
**Dominant side, n (%)**			
	Left	0 (0)	
	Right	20 (100)	

### Concurrent Validity of Isokinetic Knee Strength Measurements

On the dominant (right) side, the correlation coefficients for peak torque of knee extension and flexion were 0.662 (*P*=.001) and 0.547 (*P*=.01), respectively, with 95% LoA ranging from −22.89 to 82.21 Nm and from −26.27 to 36.01 Nm, respectively. Specifically, there was a strong correlation for peak torque during knee extension and a moderate correlation for peak torque during knee flexion. On the nondominant (left) side, the correlation coefficients for peak torque of knee extension and flexion were 0.768 (*P*<.001) and 0.347 (*P*=.12), respectively, with 95% LoA ranging from −24.60 to 75.65 Nm and from −2.40 to 75.02 Nm, respectively. The peak torque of knee extension exhibited a strong correlation, while that of knee flexion showed a weak correlation. These results are demonstrated in the scatter plot (Figure 2) and the Bland-Altman plot assessing agreement between the 2 devices (Figure 3). Detailed results of the concurrent validity analysis of isokinetic knee strength measurements are shown in Table 2.

**Figure 2:**
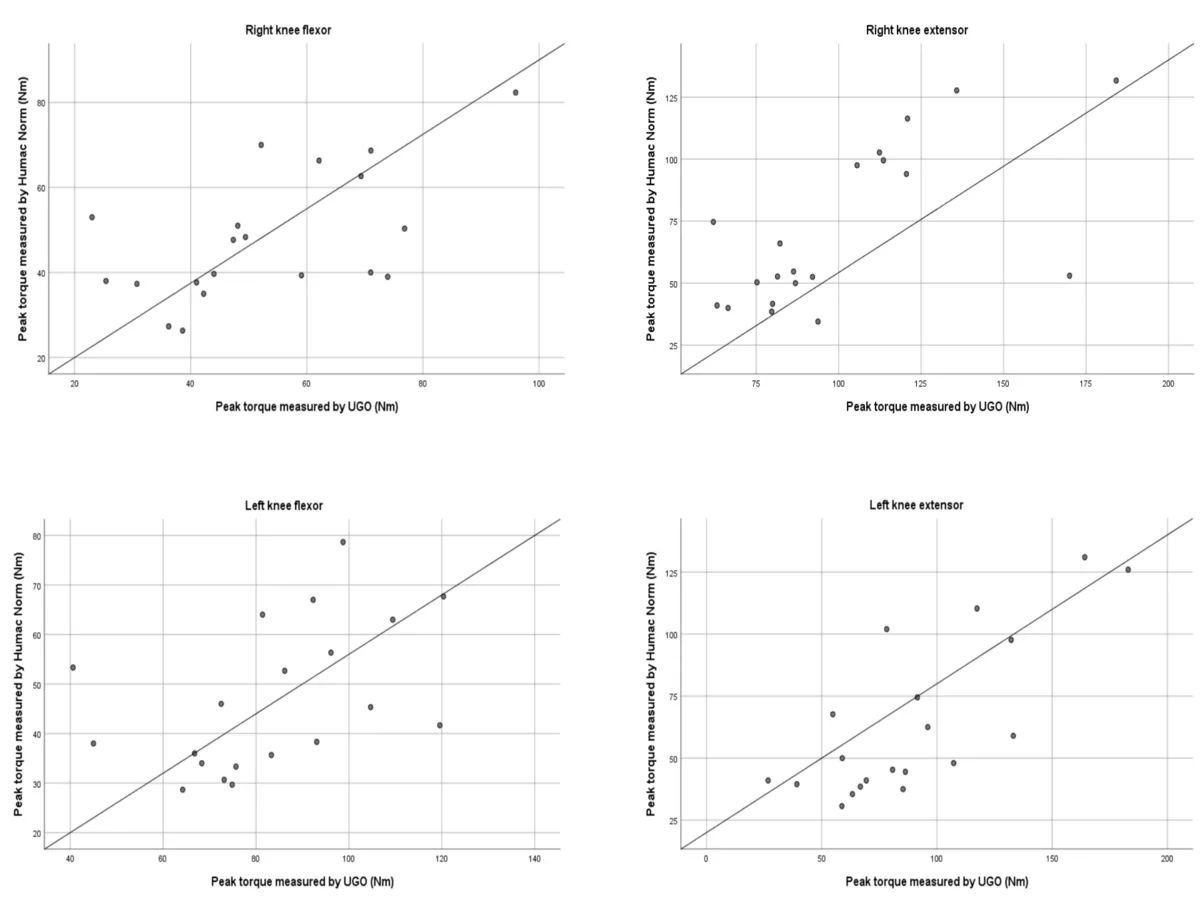
Scatter plot of knee isokinetic peak torque measured by the UGO exoskeleton and Humac Norm isokinetic dynamometer.

**Figure 3:**
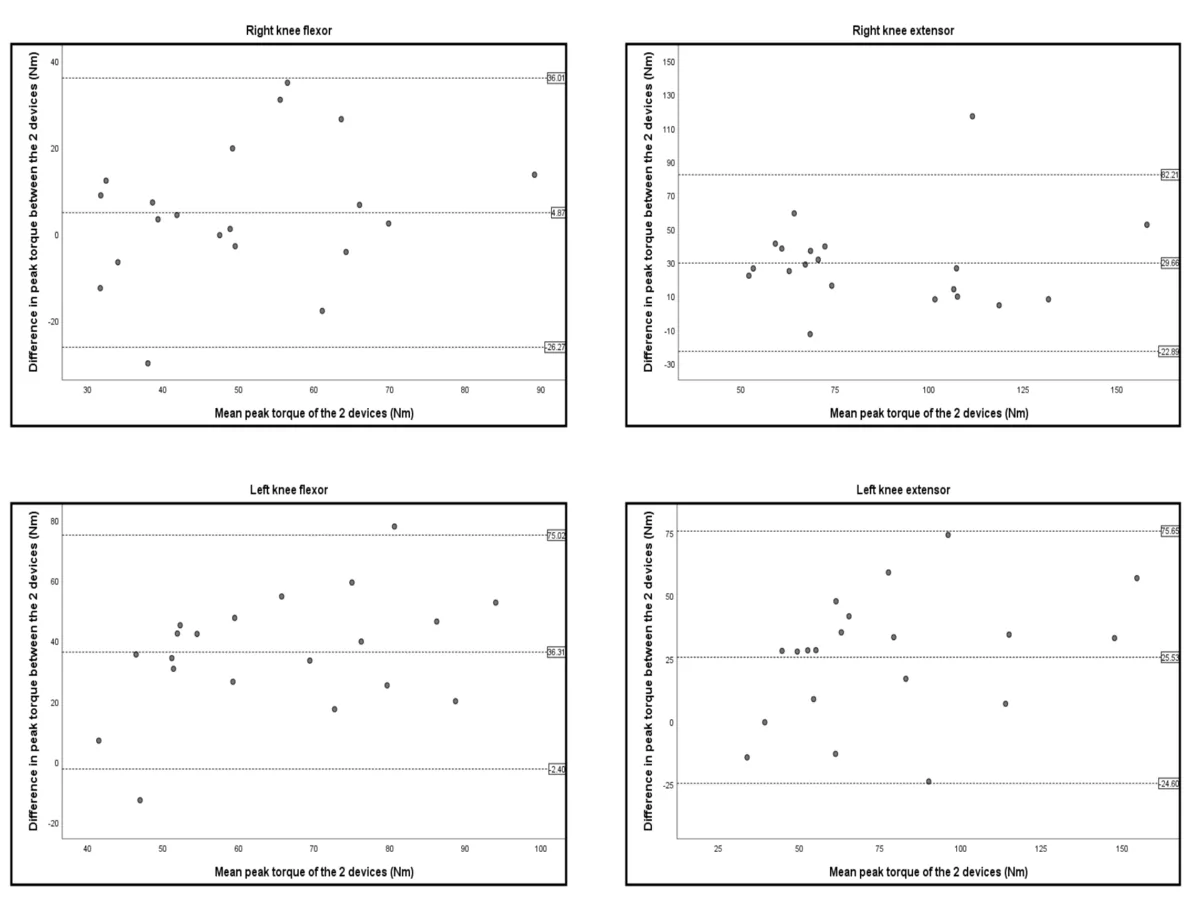
Bland-Altman plot of knee isokinetic peak torque measured by the UGO exoskeleton and Humac Norm isokinetic dynamometer.

**Table 2:** Concurrent validity of 2 isokinetic dynamometers for measuring peak torque during isokinetic knee strength testing (N=20).

Variables		Peak torque (Nm), mean (SD)			Bias (Nm; 95% limits of agreement^a^)		*r*		*P* value^b^
		UGO exoskeleton	Humac Norm						
**Dominant side**									
	Extensor	101.14 (32.46)	71.31 (31.09)	4.87 (−22.89 to 82.21)		0.662		*.001*	
	Flexor	52.89 (18.64)	49.36 (16.03)	29.66 (−26.27 to 36.01)		0.547		*.01*	
**Nondominant side**									
	Extensor	89.39 (39.11)	64.44 (31.12)	36.31 (−24.60 to 75.65)		0.768		*<.001*	
	Flexor	83.02 (21.16)	49.21 (17.71)	25.53 (−2.40 to 75.02)		0.347		.12	

^a^Bland-Altman means bias (UGO–Humac Norm) with 95% limits of agreement.

^b^Italicized values indicate significant correlations (*P*<.05).

### Concurrent Validity of Isokinetic Hip Strength Measurements

The correlation coefficients for peak torque of hip extension were 0.337 (*P*=.14) and 0.391 (*P*=.20) on the right and left sides, respectively, with 95% LoA ranging from −51.57 to 76.80 Nm and from −28.19 to 79.59 Nm, respectively, and showed no significant correlation. The correlation coefficients for peak torque of hip flexion were 0.615 (*P*=.003) and 0.443 (*P*=.04), on the right and left sides, respectively, with 95% LoA ranging from −15.52 to 86.57 Nm and from −12.53 to 98.50 Nm, respectively. The peak torque of right hip flexion exhibited a strong correlation, while that of left hip flexion showed a moderate correlation. These results are demonstrated in the scatter plot (Figure 4) and the Bland-Altman plot assessing agreement between the 2 devices (Figure 5). Detailed results of the concurrent validity analysis of isokinetic hip strength measurements are shown in Table 3.

**Figure 4:**
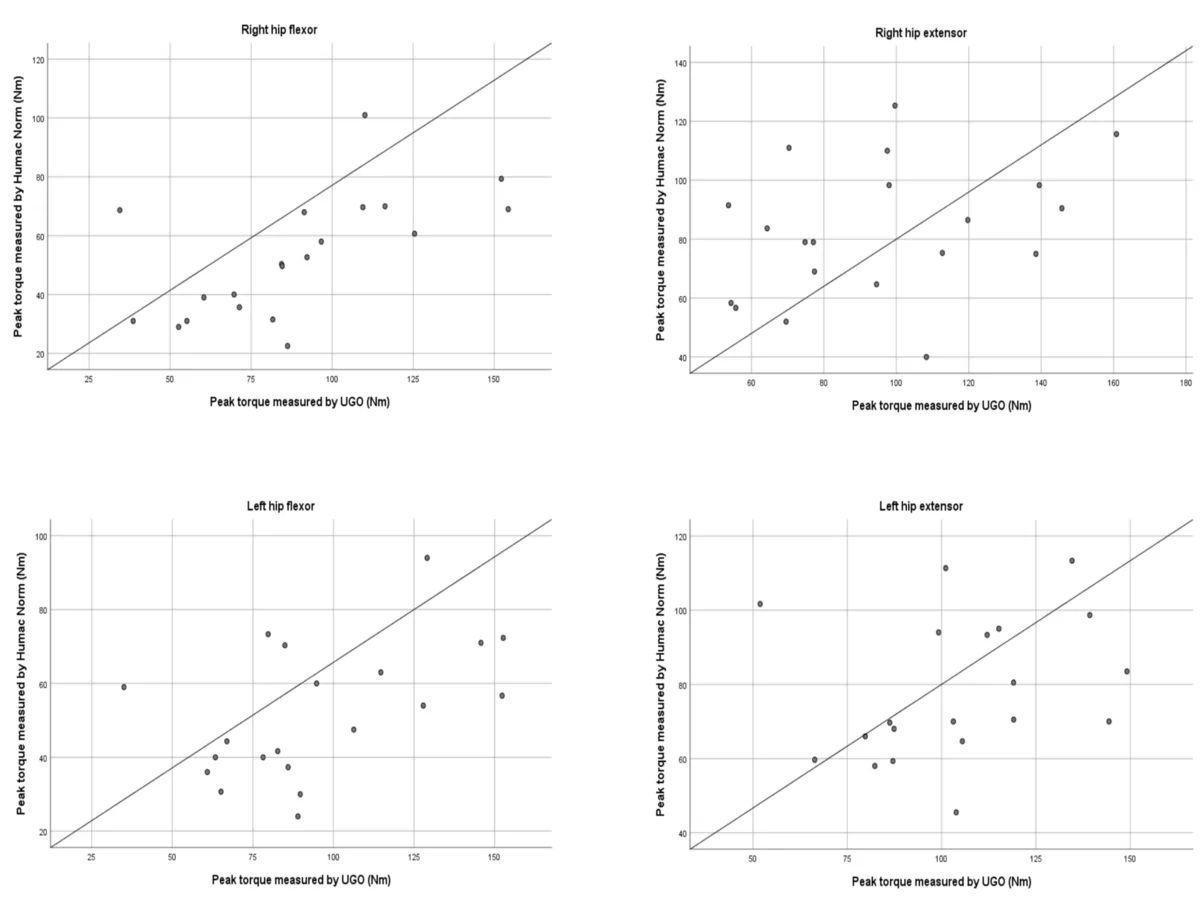
Scatter plot of hip isokinetic peak torque measured by the UGO exoskeleton and Humac Norm isokinetic dynamometer.

**Figure 5:**
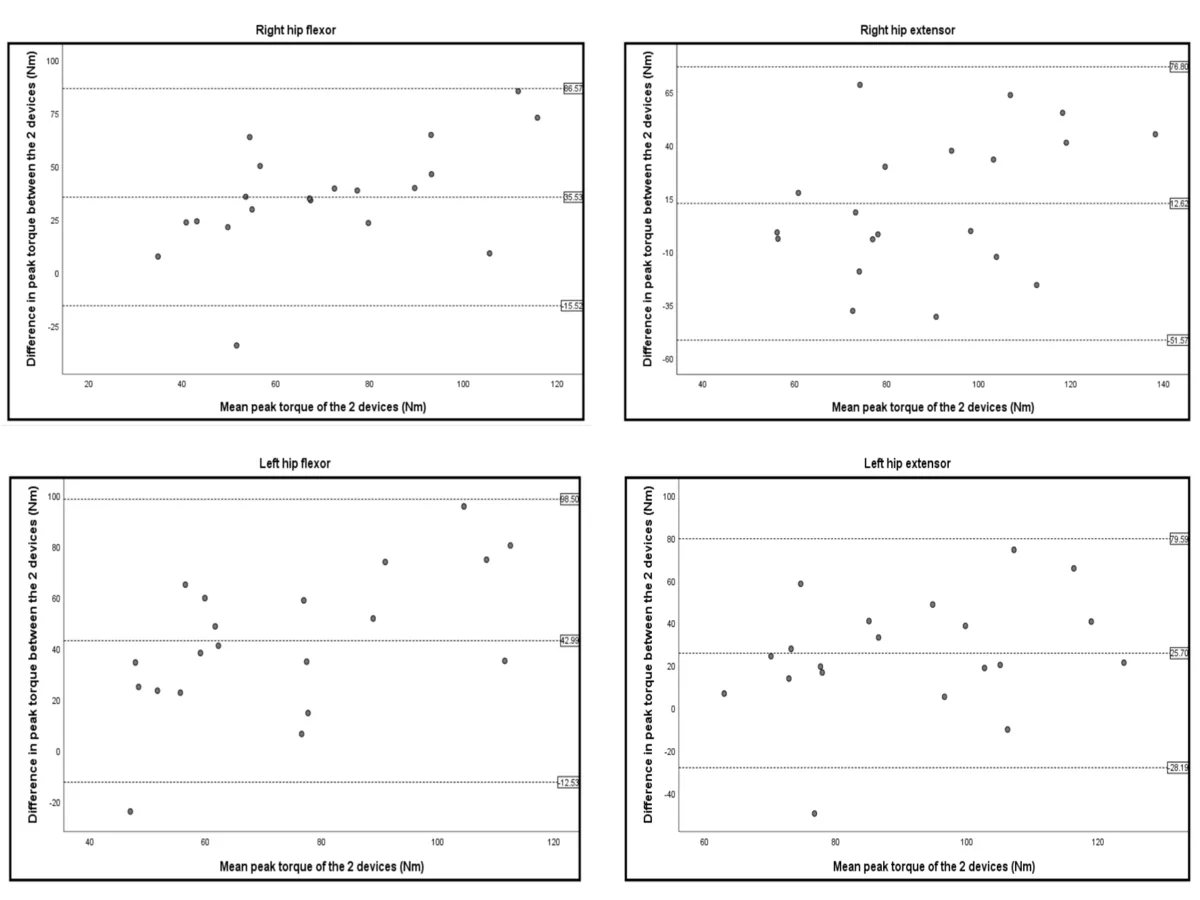
Bland-Altman plot of hip isokinetic peak torque measured by the UGO exoskeleton and Humac Norm isokinetic dynamometer.

**Table 3:** Concurrent validity of 2 isokinetic dynamometers for measuring peak torque during isokinetic hip strength testing (N=20).

Variables		Peak torque (Nm), mean (SD)		Bias (Nm; 95% limits of agreement^a^)	*r*	*P* value^b^
		UGO exoskeleton	Humac Norm			
**Dominant side**						
	Extension	95.61 (31.65)	82.80 (22.13)	35.53 (−51.57 to 76.80)	0.337	.14
	Flexion	88.17 (32.29)	53.06 (20.02)	12.62 (−15.52 to 86.57)	0.615	*.003*
**Nondominant side**						
	Extension	103.70 (25.25)	80.62 (20.77)	42.99 (−28.19 to 79.59)	0.391	.20
	Flexion	94.24 (32.20)	53.29 (18.31)	25.70 (−12.53 to 98.50)	0.443	*.04*

^a^Bland-Altman means bias (UGO–Humac Norm) with 95% limits of agreement.

^b^Italicized values indicate significant correlations (*P*<.05).

## Discussion

### Principal Findings

To the best of our knowledge, this study represents the first attempt to investigate the concurrent validity of isokinetic muscle strength testing in the context of lower limb exoskeleton rehabilitation robots. In this cross-sectional validation study, the UGO exoskeleton showed moderate to strong concurrent validity—with significant correlations of at least moderate strength and Bland-Altman agreement—for the bilateral knee extensors and the hip flexors, but not for the left knee flexors or bilateral hip extensors, which showed nonsignificant associations and greater systematic bias. Across all muscle actions, the UGO systematically overestimated peak torque relative to the Humac Norm.

Previous studies comparing torque measurements obtained with hand-held dynamometers and isokinetic dynamometers have found that tests of the knee extensor and flexor muscles show moderate to strong correlations [32], while tests of internal and external rotation of the shoulder joint show weak correlations [33]. O’Neill et al [10] found that the C-station (Fysiometer) appeared to provide valid measurements compared with an isokinetic dynamometer for plantar flexor isometric strength. However, these studies only compared devices with an isokinetic dynamometer during isometric contraction tests. Our study compared 2 devices during isokinetic knee and hip strength testing. We found that the UGO showed strong correlations for both knee extensor torque measurements, a moderate correlation for right knee flexor torque, and a nonsignificant correlation for left knee flexor torque compared to the reference standard, the Humac Norm. In hip testing, the correlation of the flexor torque values was stronger than that of the extensor torque values on both sides. The underlying reasons for these differences included 4 factors. First, the test positions differed. During the UGO isokinetic strength test, the knee and hip flexion and extension muscles were tested in the upright position. In contrast, during the Humac Norm isokinetic strength test, the knee was positioned in the seated posture, while the hip was placed in the supine position. Diverse test positions can influence force production, thereby leading to varying torque values. During the upright position test, participants consistently reported that exerting force for hip flexion combined with knee extension required less effort than hip extension combined with knee flexion. Second, the torque signal acquisition and calculation formulas differed between the 2 devices. Despite the sampling rate of both torque sensors being 100 Hz [23,34], the torque sensor of the UGO exoskeleton has a measurement range of 150 Nm, a resolution of 0.05 Nm, and a force-sensing accuracy of 0.3% [23]. The resolution and accuracy of the Humac Norm isokinetic dynamometer force sensor have not been reported in the existing literature, but Habets et al [35] reported that the Humac Norm dynamometer showed good to excellent reliability in strength tests of the knee and shoulder joints. The disparities in the mechanical structure and software of the 2 devices may likewise affect the calculation of the lever arm and the torque value of the generated force [27]. As torque is obtained by multiplying the measured force by the perpendicular lever-arm length (τ=F×d⊥), such device-related discrepancies have been shown to produce systematic torque differences of around 8 Nm between hand-held and isokinetic dynamometers [36]. Third, the number of torque sensors differed between the 2 devices. The UGO dynamometer has 4 torque sensors, and the Humac Norm has only 1 sensor. We hypothesize that the presence of numerous force sensors may increase the potential for error associated with torque calculation [37]. Finally, factors related to the patient’s maximum voluntary force contraction may have contributed to the differences. In knee testing, the correlation coefficient for peak hamstring torque appeared to be lower than that for peak quadriceps torque [25]. This finding may be partly explained by the characteristically lower test-retest reliability of hamstring strength measurements relative to those of the quadriceps [38].

In hip testing, the complexity and standardization of hip joint testing present additional challenges. Hip movements are more difficult to isolate and standardize than knee tasks because of greater degrees of freedom, pelvic tilt variability, and compensatory trunk or lumbar involvement. The UGO, as a functional exoskeleton, may capture more multijoint, closed-chain dynamics, while Humac Norm emphasizes open-chain, single-plane isokinetic testing, leading to divergent torque profiles. Hip extension involves strong gluteal or hamstring recruitment with potential antagonist cocontraction (eg, flexors and abdominals), which exoskeletons may detect differently from laboratory dynamometers. Participants may unconsciously alter their movement strategies (eg, trunk leaning) on the UGO, inflating or deflating measured torque relative to Humac Norm. Interestingly, we found that the measurement of peak torque exhibited significantly higher correlation coefficients for the anterior muscle group than the posterior muscle group.

For patients, a significant inconvenience arises from standard isokinetic muscle strength testing, which requires repositioning the body when assessing multiple joints in the lower limbs. Although the UGO’s isokinetic muscle strength test results **e**xhibit certain discrepancies compared to those of the Humac Norm reference standard, it offers superior clinical convenience. In particular, the isokinetic muscle strength testing function of the UGO device enables therapists to quantitatively assess changes in patients’ lower limb function during clinical treatment and across treatment courses (preintervention and postintervention). The exoskeleton robot establishes a closed-loop rehabilitation training protocol for patients, allowing the rehabilitation therapist to adjust the training program based on the results of muscle strength testing.

Overall, the UGO’s isokinetic muscle strength test is more convenient and maneuverable than traditional isokinetic dynamometers. This device not only reduces the operational requirements of therapist-operated devices and saves time, but it also alleviates patient discomfort by minimizing repositioning—especially for those with mobility difficulties. Therefore, the isokinetic muscle strength assessment capability of the UGO device enables therapists to evaluate lower limb motor function and implement targeted treatment strategies more effectively in clinical rehabilitation settings.

### Limitations

This study has several limitations. First, the inherent hardware differences between the devices inevitably lead to discrepancies in test posture and lever-arm length. Second, device-specific error sources for the UGO include reliance on estimated segment lengths for lever-arm or torque computation, the distributed 4-sensor architecture, and a proprietary, nontransparent torque calculation algorithm; for the reference device, fatigue status before Humac Norm testing was assessed only subjectively, which may partly explain the relatively lower recorded torques. Third, the relatively small sample size in this study may limit the statistical power of the analyses and preclude definitive conclusions. Fourth, only healthy young adults were studied; findings cannot be extrapolated to patients with neurological impairment, in whom spasticity, weakness, altered tone, impaired trunk control, and reduced range of motion may alter both torque magnitude and device-reference agreement. Finally, as the device testing order was fixed, potential order effects（learning or fatigue）cannot be fully excluded, although the 24-hour interdevice interval and activity restriction were intended to mitigate them.

### Conclusions

In healthy young adults, the UGO exoskeleton demonstrated moderate to strong concurrent validity for peak torque of the bilateral knee extensors and the hip flexors, but not for the nondominant knee flexors and bilateral hip extensors, and it systematically overestimated peak torque relative to the Humac Norm. The device may serve as a convenient torque assessment tool for selected muscle actions; however, its use for the knee flexors and hip extensors is not yet supported, and validation in the intended clinical population is required in future studies.

## Abbreviations

LoA: limits of agreement

## Data Availability

The data are available from the corresponding author upon reasonable request.

## References

[1] Grimmer M, Riener R, Walsh CJ, Seyfarth A (2019). Mobility related physical and functional losses due to aging and disease - a motivation for lower limb exoskeletons. J Neuroeng Rehabil.

[2] Wang D, Wu Y, Yu H (2024). State of the art of brain function detection technologies in robot-assisted lower limb rehabilitation. Brain Connect.

[3] Tang C, Zhang Y, Zhou T, Yin R, Zhang L, Ren C, Gong Z, Wu Y, Chen Y, Zhu Y, Song P, Chen W, Wang H (2026). Effects of lower limb exoskeleton robot-assisted rehabilitation on motor function, activities of daily living, and electromyography in hemiplegic stroke: a randomized controlled trial. BMC Neurol.

[4] Chen YN, Wu YN, Yang BS (2023). The neuromuscular control for lower limb exoskeleton- a 50-year perspective. J Biomech.

[5] Flansbjer UB, Downham D, Lexell J (2006). Knee muscle strength, gait performance, and perceived participation after stroke. Arch Phys Med Rehabil.

[6] Muehlbauer T, Gollhofer A, Granacher U (2015). Associations between measures of balance and lower-extremity muscle strength/power in healthy individuals across the lifespan: a systematic review and meta-analysis. Sports Med.

[7] Tayfur B, Johnson AK, Palmieri-Smith RM (2025). Compromised quadriceps and hamstring force control, not maximal strength, is associated with gait biomechanics at 9 months following anterior cruciate ligament reconstruction. Knee Surg Sports Traumatol Arthrosc.

[8] Hsu WH, Hsu WB, Lin ZR, Chang SH, Fan CH, Kuo LT, Hsu WW (2023). Effects of 24 weeks of a supervised walk training on knee muscle strength and quality of life in older female total knee arthroplasty: a retrospective cohort study. Healthcare (Basel).

[9] Bohannon RW (2019). Considerations and practical options for measuring muscle strength: a narrative review. Biomed Res Int.

[10] O'Neill S, Weeks A, Nørgaard JE, Jorgensen MG (2023). Validity and intrarater reliability of a novel device for assessing plantar flexor strength. PLoS One.

[11] Prasad R, El-Rich M, Awad MI, Hussain I, Jelinek HF, Huzaifa U, Khalaf K (2022). A framework for determining the performance and requirements of cable-driven mobile lower limb rehabilitation exoskeletons. Front Bioeng Biotechnol.

[12] Huo C, Shao G, Chen T, Li W, Wang J, Xie H, Wang Y, Li Z, Zheng P, Li L, Li L (2024). Effectiveness of unilateral lower-limb exoskeleton robot on balance and gait recovery and neuroplasticity in patients with subacute stroke: a randomized controlled trial. J Neuroeng Rehabil.

[13] Zhang Q, Chen Y, Lewek M, Si J, Huang H (2026). Towards adaptive optimal personalization control of robotic hip exoskeleton assistance for individuals with paretic stroke. IEEE Trans Biomed Eng.

[14] Yang J, Gong Y, Yu L, Peng L, Cui Y, Huang H (2023). Effect of exoskeleton robot-assisted training on gait function in chronic stroke survivors: a systematic review of randomised controlled trials. BMJ Open.

[15] Zhang Y, Liu X, Qiao X, Fan Y (2023). Characteristics and emerging trends in research on rehabilitation robots from 2001 to 2020: bibliometric study. J Med Internet Res.

[16] Zhang Y, Zhao W, Wan C, Wu X, Huang J, Wang X, Huang G, Ding W, Chen Y, Yang J, Su B, Xu Y, Zhou Z, Zhang X, Miao F, Li J, Li Y (2024). Exoskeleton rehabilitation robot training for balance and lower limb function in sub-acute stroke patients: a pilot, randomized controlled trial. J Neuroeng Rehabil.

[17] Liu H, Zhu C, Zhou Z, Dong Y, Meng W, Liu Q (2024). Synergetic gait prediction and compliant control of SEA-driven knee exoskeleton for gait rehabilitation. Front Bioeng Biotechnol.

[18] Riener R, Nef T, Colombo G (2005). Robot-aided neurorehabilitation of the upper extremities. Med Biol Eng Comput.

[19] Domingo A, Lam T (2014). Reliability and validity of using the Lokomat to assess lower limb joint position sense in people with incomplete spinal cord injury. J Neuroeng Rehabil.

[20] Xu D, Kang SH, Lee SJ, Oppizzi G, Zhang LQ (2024). Multi-joint assessment of proprioception impairments poststroke. Arch Phys Med Rehabil.

[21] De Oliveira AC, Sulzer JS, Deshpande AD (2021). Assessment of upper-extremity joint angles using Harmony exoskeleton. IEEE Trans Neural Syst Rehabil Eng.

[22] Zhang S, Fan L, Ye J, Chen G, Fu C, Leng Y (2023). An intelligent rehabilitation assessment method for stroke patients based on lower limb exoskeleton robot. IEEE Trans Neural Syst Rehabil Eng.

[23] Yang W, Zhang J, Zhang S, Yang C (2020). Lower limb exoskeleton gait planning based on crutch and human-machine foot combined center of pressure. Sensors.

[24] Zhao JL, Xie H, Chen PM, Ng SS, Wang CH (2026). Intra-tester and inter-tester reliability of maximum isokinetic hip and knee strength measurements with the exoskeleton in healthy young adults. Front Sports Act Living.

[25] Whiteley R, Jacobsen P, Prior S, Skazalski C, Otten R, Johnson A (2012). Correlation of isokinetic and novel hand-held dynamometry measures of knee flexion and extension strength testing. J Sci Med Sport.

[26] de Araujo Ribeiro Alvares JB, Rodrigues R, de Azevedo Franke R, da Silva BG, Pinto RS, Vaz MA, Baroni BM (2015). Inter-machine reliability of the Biodex and Cybex isokinetic dynamometers for knee flexor/extensor isometric, concentric and eccentric tests. Phys Ther Sport.

[27] Whinton AK, Thompson KM, Power GA, Burr JF (2018). Testing a novel isokinetic dynamometer constructed using a 1080 Quantum. PLoS One.

[28] Fouasson-Chailloux A, Daley P, Menu P, Gadbled G, Bouju Y, Gautier G, Pomares G, Dauty M (2022). Use of hand hydraulic dynamometers as an overall evaluation of the upper-limb weakness in patients with thoracic outlet syndrome. Front Neurol.

[29] Feiring DC, Ellenbecker TS, Derscheid GL (1990). Test-retest reliability of the biodex isokinetic dynamometer. J Orthop Sports Phys Ther.

[30] Jaric S (2002). Muscle strength testing: use of normalisation for body size. Sports Med.

[31] Post MW (2016). What to do with "moderate" reliability and validity coefficients?. Arch Phys Med Rehabil.

[32] Muff G, Dufour S, Meyer A, Severac F, Favret F, Geny B, Lecocq J, Isner-Horobeti ME (2016). Comparative assessment of knee extensor and flexor muscle strength measured using a hand-held vs. isokinetic dynamometer. J Phys Ther Sci.

[33] Loepfe JG, Nagelli CV, Larson DR, Tagliero AJ, Cherny CE, Johnson AJ (2025). Reliability and validity of measuring shoulder internal and external rotation strength in volleyball specific positions. Int J Sports Phys Ther.

[34] Luo H, Xiong Y, Zhu M, Wei X, Tao X (2024). Integrated wearable system for monitoring skeletal muscle force of lower extremities. Sensors (Basel).

[35] Habets B, Staal JB, Tijssen M, van Cingel R (2018). Intrarater reliability of the Humac NORM isokinetic dynamometer for strength measurements of the knee and shoulder muscles. BMC Res Notes.

[36] Garcia MA, Fonseca DS, Souza VH (2021). Handheld dynamometers for muscle strength assessment: pitfalls, misconceptions, and facts. Braz J Phys Ther.

[37] Edouard P, Codine P, Samozino P, Bernard PL, Hérisson C, Gremeaux V (2013). Reliability of shoulder rotators isokinetic strength imbalance measured using the Biodex dynamometer. J Sci Med Sport.

[38] Li RC, Wu Y, Maffulli N, Chan KM, Chan JL (1996). Eccentric and concentric isokinetic knee flexion and extension: a reliability study using the Cybex 6000 dynamometer. Br J Sports Med.

